# Multifunctional Polyimide for Packaging and Thermal Management of Electronics: Design, Synthesis, Molecular Structure, and Composite Engineering

**DOI:** 10.3390/nano15151148

**Published:** 2025-07-24

**Authors:** Xi Chen, Xin Fu, Zhansheng Chen, Zaiteng Zhai, Hongkang Miu, Peng Tao

**Affiliations:** 1 State Key Laboratory of Metal Matrix Composites, School of Materials Science and Engineering, Shanghai Jiao Tong University, 800 Dong Chuan Road, Shanghai 200240, China; chenxi1@sjtu.edu.cn; 2 Shanghai Institute of Satellite Engineering, 3666 Yuanjiang Road, Minhang District, Shanghai 201109, China; boston_z@sina.com (Z.Z.);; 3 Shanghai Academy of Spaceflight Technology, 3888 Yuanjiang Road, Minhang District, Shanghai 201109, China; 4 National Engineering Research Center of Special Equipment and Power System for Ship and Marine Engineering, Shanghai 200030, China

**Keywords:** polyimides, surface modification, dielectric properties, nanocomposites, packaging and thermal management

## Abstract

Polyimide, a class of high-performance polymers, is renowned for its exceptional thermal stability, mechanical strength, and chemical resistance. However, in the context of high-integration and high-frequency electronic packaging, polyimides face critical challenges including relatively high dielectric constants, inadequate thermal conductivity, and mechanical brittleness. Recent advances have focused on molecular design and composite engineering strategies to address these limitations. This review first summarizes the intrinsic properties of polyimides, followed by a systematic discussion of chemical synthesis, surface modification approaches, molecular design principles, and composite fabrication methods. We comprehensively examine both conventional polymerization synthetic routes and emerging techniques such as microwave-assisted thermal imidization and chemical vapor deposition. Special emphasis is placed on porous structure engineering via solid-template and liquid-template methods. Three key modification strategies are highlighted: (1) surface modifications for enhanced hydrophobicity, chemical stability, and tribological properties; (2) molecular design for optimized dielectric performance and thermal stability; and (3) composite engineering for developing high-thermal-conductivity materials with improved mechanical strength and electromagnetic interference (EMI) shielding capabilities. The dielectric constant of polyimide is reduced while chemical stability and wear resistance can be enhanced through the introduction of fluorine groups. Ultra-low dielectric constant and high-temperature resistance can be achieved by employing rigid monomers and porous structures. Furthermore, the incorporation of fillers such as graphene and boron nitride can endow the composite materials with high thermal conductivity, excellent EMI shielding efficiency, and improved mechanical properties. Finally, we discuss representative applications of polyimide and composites in electronic device packaging, EMI shielding, and thermal management systems, providing insights into future development directions.

## 1. Introduction

With the rapid development of electronic devices, integrated circuits, and mobile equipment, conventional thermal management components like copper heat pipes face challenges in meeting the miniaturization requirements of thermal management systems in portable devices such as smartphones, tablets, smartwatches, and wearables due to their large volume, heavy weight, and difficult processing. Polymer-based packaging and thermal management materials have been widely adopted in various electronics owing to their lightweight nature, flexibility, and processability. Polyimide is a type of polymer characterized by the presence of imide rings in its backbone, typically formed through the condensation of dianhydride and diamine. Its unique combination of properties, including high thermal stability, excellent mechanical strength, and outstanding chemical resistance, stems from rigid aromatic structures and strong intermolecular interactions [1]. These advantages make polyimides particularly suitable for weight-sensitive applications like flexible electronics [2,3]. In 1908, M.T. Bogert and R.R. Renshaw [4] first synthesized polyimide. However, due to its high melting point and poor processability, it did not experience rapid development or applications at that time. The true advancement of polyimide materials began with the DuPont Company in the United States. In 1961, DuPont produced Kapton^®^ polyimide film, followed by Vespel^®^ polyimide film in 1964. Subsequently, companies in the United States, Japan, France, and other countries successively invested in the research, development, and production of polyimides, synthesizing a wide variety of polyimide products such as fibers, films, adhesives, and polyimide-based composites. This marked the beginning of a rapid development period for polyimide research.

**Figure 1:**
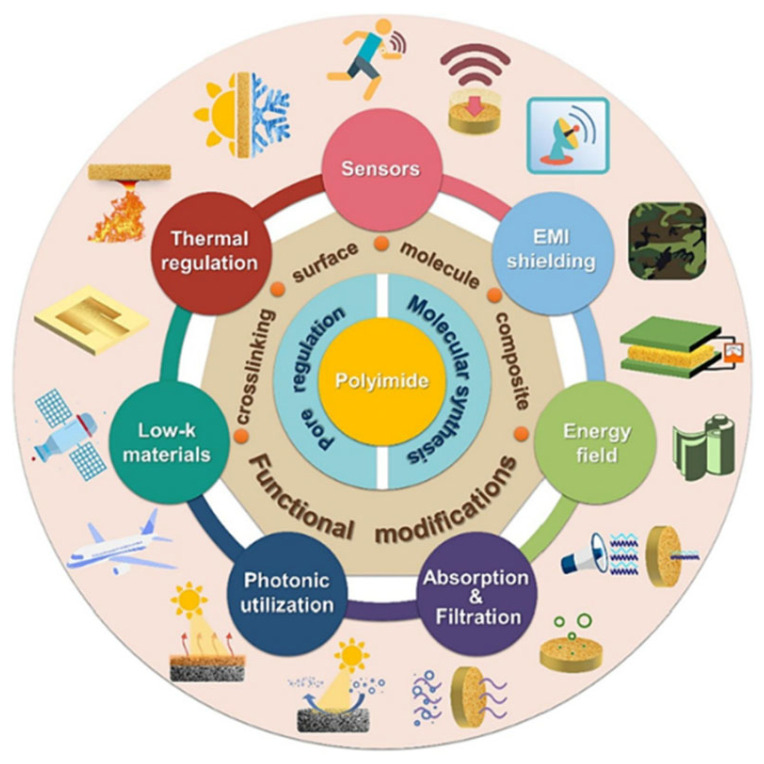
Diverse application of polyimides (Figure 1 reprinted/adapted with permission from Ref. [5]. 2024, Elsevier).

The increasing operation power density and integration level of electronic devices and systems raise harsh requirements on heat-transfer and dielectric properties of polyimides. However, the thermal conductivity of pure polyimide is usually low, generally in the range of 0.1–0.3 W/m K, which cannot meet the thermal conduction demands. Meanwhile, electromagnetic (EMI) shielding has become an indispensable requirement for advanced electronic packaging because it can help avoid signal distortion and enable stable operation under complex electromagnetic environments. But pure polyimide has relatively poor EMI shielding properties, typically with a shielding effectiveness (SE) of only around 0–10 dB in the high-frequency range due to a lack of free electrons or conductive networks to block or absorb electromagnetic waves. Fortunately, recent studies indicated that the incorporation of high thermal conductivity fillers (e.g., graphene) could enable polyimide composites to achieve significantly enhanced thermal conductivity while achieving excellent EMI shielding performance. In recent years, the development of novel polyimide derivatives and composites has expanded their applications from packaging and thermal management of electronics into emerging fields such as flexible electronics, 3D printing, and biomedical fields (Figure 1) [5,6].

In this work, we review recent progress in the design, preparation, and property tuning of polyimide materials and composites for packaging and thermal management of electronics. We firstly introduce both conventional synthesis methods and emerging preparation techniques for both nonporous and porous polyimides. We then highlight representative surface modification strategies to enhance hydrophobicity, chemical resistance and friction. In terms of development of functional polyimides and composites, we introduce advanced molecular structure design to achieve lower dielectric constant and high thermal conductivity of polyimide, and discuss the composite engineering to prepare thermal conductive, mechanically robust and EMI-shielding polyimide composites. We briefly introduce their corresponding applications in electronic device packaging and thermal management domains as well as performance enhancements. Finally, we also summarize recent advancements in this field and propose prospects for future development.

## 2. Fabrication and Surface Modification Strategies

### 2.1. Polyimide Synthesis Methods

**Figure 2:**
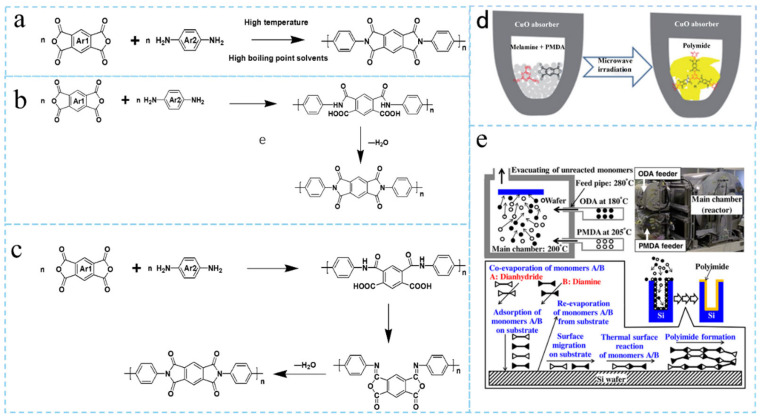
Schematic diagrams of polyimide synthesis methods: (**a**) one-step method, (**b**) two-step method, (**c**) three-step method, (**d**) microwave-assisted imidization (Figure 2d reprinted/adapted with permission from Ref. [10]. 2015, Royal Society of Chemistry), and (**e**) the vapor deposition method (Figure 2e reprinted/adapted with permission from Ref. [11]. 2020, Elsevier).

As shown in Figure 2a–c, polyimide synthesis mainly involves three process routes: one-step [7], two-step [8], and three-step methods [9].

In the one-step method, diamine and dianhydride are dissolved in high-boiling-point solvents (e.g., m-cresol, NMP) for direct polycondensation at elevated temperatures, simultaneously achieving imidization and chain extension. While operationally simple, it requires dehydrating agents or azeotropic water removal to prevent dianhydride deactivation. This method directly polymerizes dianhydride and diamine monomers in high-boiling solvents to produce polyimides, showing its greatest advantages in simple, high-efficiency processing. It eliminates intermediate steps (e.g., synthesis of polyamic acid), shortens production cycles, and reduces equipment complexity. Since imide rings form directly, the product contains no polyamic acid residues, avoiding hydrolysis or degradation risks while achieving higher purity and stability. Moreover, its flexible solvent selection (e.g., m-cresol) makes it particularly suitable for rigid monomers (e.g., biphenyl dianhydride), directly yielding products (T_g_ > 400 °C) with high thermal stability that are ideal for aerospace high-temperature materials.

The two-step method, currently the most widely used process, first conducts solution polycondensation in aprotic polar solvents (e.g., N-methylpyrrolidone,N,N-dimethylformamide) to form polyamic acid (PAA) precursors, followed by thermal (gradient heating to 300 °C) or chemical imidization (using dehydrating agents like acetic anhydride/triethylamine), with molecular weight controllable via the Carothers equation. Its core advantage lies in superior processability. Adjustable PAA solution viscosity facilitates film casting, spinning, or coating, making it widely applicable in flexible circuit boards (e.g., Kapton films). Synthesis of PAA at room temperature avoids high-temperature side reactions, yielding more uniform molecular weight distribution, while subsequent thermal or chemical imidization enables precise performance control. Additionally, the PAA stage readily accommodates fillers or comonomers for functional modifications (e.g., enhanced mechanical or dielectric properties). Despite PAA suffering from moisture sensitivity and poor storage stability, the two-step method remains dominant in fabricating polyimides for electronic packaging and display substrates.

The three-step method enhances the two-step process by incorporating chemical imidization, further improving the imidization degree and controllability over molecular weight. Its primary advantages include reduced material defects and improved uniformity. Gradual imidization lowers intramolecular chain stress, minimizing the formation of micropores and cracks while increasing the tensile strength of films and ensuring more complete solvent removal. Moreover, by controlling pre-imidization conditions, it precisely regulates crystallinity and pore structure, making it suitable for thick-film preparation (preventing warpage) or functional films (e.g., lithium battery separators). Despite complex procedures and higher costs, the three-step method proves indispensable for defect-sensitive applications like high-frequency substrates and new energy devices.

Each synthesis method has distinct characteristics: the one-step method offers simplicity but challenges in molecular weight control; the two-step method balances process complexity and product performance; while the three-step method delivers superior performance at the cost of greater process complexity.

Moreover, recent years have witnessed the emergence of novel synthesis technologies. As shown in Figure 2d, microwave-assisted imidization [10] leverages the selective heating characteristics of microwaves to significantly enhance reaction rates and reduce energy consumption, shortening conventional thermal imidization processes from hours to minutes. Meanwhile, vapor deposition polymerization (VDP) [11,12] overcomes the limitations of solution-based methods by directly polymerizing vaporized monomers on substrates (Figure 2e), enabling precise fabrication of ultrathin polyimide films. This approach is particularly suitable for microelectronic applications that require ultrathin insulating layers. For instance, Fukushima et al. [12] successfully prepared 500 nm-thick polyimide films via solvent-free VDP of pyromellitic dianhydride and 4,4′-oxydianiline at 200 °C. These innovative techniques not only expand the processing window for polyimides but also provide new avenues for performance modulation.

### 2.2. Surface Modification

Surface modification techniques can effectively tailor the surface characteristics of polyimide materials through physical, chemical, or hybrid treatment approaches. These techniques can significantly enhance wettability, chemical stability, tribological properties, and bring extra functionalities to meet specific application requirements.

#### 2.2.1. Hydrophobicity Enhancement

Conventional polyimide materials still exhibit certain hygroscopicity, where moisture penetration may lead to deteriorated dielectric properties, metal line corrosion, and interfacial delamination, consequently compromising the long-term reliability of electronic devices. Therefore, enhancing the hydrophobicity of polyimides is crucial for their application in advanced electronic packaging. Improved hydrophobicity not only reduces moisture absorption but also enhances chemical corrosion resistance and interfacial adhesion strength, thereby meeting the stringent material requirements for high-density integrated circuits, flexible electronics, and cutting-edge packaging technologies. Two-dimensional (2D) MXenes (transition metal carbides/nitrides) significantly enhance the hydrophobic properties of polymers as nanofillers by virtue of their unique layered structure and tunable interfacial chemistry. Wang et al. [13] fabricated lightweight and hydrophobic polyimide/MXene 3D architectures via freeze-drying of MXene/polyimide suspensions, achieving an intrinsic water contact angle of 119° without surface modification. The MXene nanosheets were encapsulated by polyimide chains through strong hydrogen bonding, while polar groups of polyimides were embedded within the internal network, thereby reducing hydrophilicity. Fluorination treatment creates low-surface-energy structures through chemical reactions to achieve hydrophobicity. Li et al. [14] developed a superhydrophobic polyimide with a water contact angle of 156° by immersing polyimide in Fluorocarbon Resin solution. Chemical vapor deposition (CVD) [15] can also introduce fluorine-containing groups, elevating contact angles beyond 120°.

Notably, constructing PAA nanofiber-based isotropic 3D networks [16] could yield super-elastic polyimide nanofiber aerogels with high hydrophobicity, which could be attributed to enhanced surface roughness from interconnected fibrous and porous architectures. Bioinspired micro/nano-structures, such as lotus-leaf-like hierarchical textures, fabricated via templating [17] or phase separation, can further enhance contact angles above 150°, achieving superhydrophobicity.

#### 2.2.2. Chemical Resistance Improvement

While polyimides exhibit exceptional chemical stability in extreme environments—a core advantage—their resistance to chemical corrosion requires further enhancement for specialized applications such as advanced semiconductor packaging. Surface modification can effectively improve its resistance to acids, alkalis, organic solvents, and other chemical media while preserving its intrinsic superior properties. For instance, silica nanoparticle/sol-modified polyimide fibers [18] yield acid- and alkali-resistant textiles. Woven from these fibers, the fabric demonstrates superhydrophobicity alongside tolerance to both alkaline and acidic solutions. Alternatively, incorporating trimethoxysilane as side groups into polyimide units, followed by hydrolysis and condensation, forms a highly crosslinked Si-O-Si network within the porous polyimide structure, significantly enhancing chemical stability [19]. Of particular note, the introduction of cyano functional groups [20] improves solubility while maintaining the inherent chemical resistance through molecular structure optimization. As listed in Table 1, the synthesis of CNFPI, CNOPI, and FPI was achieved using 2,5-diaminobenzonitrile, 4,4′-(hexafluoroisopropylidene)diphthalic anhydride, and 4,4′-oxydiphthalic anhydride (ODA), respectively. Subsequently, CNFPI and CNOPI were thermally cured at elevated temperatures to yield cross-linked CCNFPI and CCNOPI.

Research results demonstrate that the introduction of cyano groups (-CN) enables the polyimides to dissolve in organic solvents at room temperature. This enhanced solubility is attributed to the large dipole moment of the cyano group, which strengthens the solvation effect with polar solvents. However, after thermal treatment, both CCNFPI and CCNOPI become insoluble in all common organic solvents, confirming that polymer cross-linking significantly improves chemical stability.

**Figure 3:**
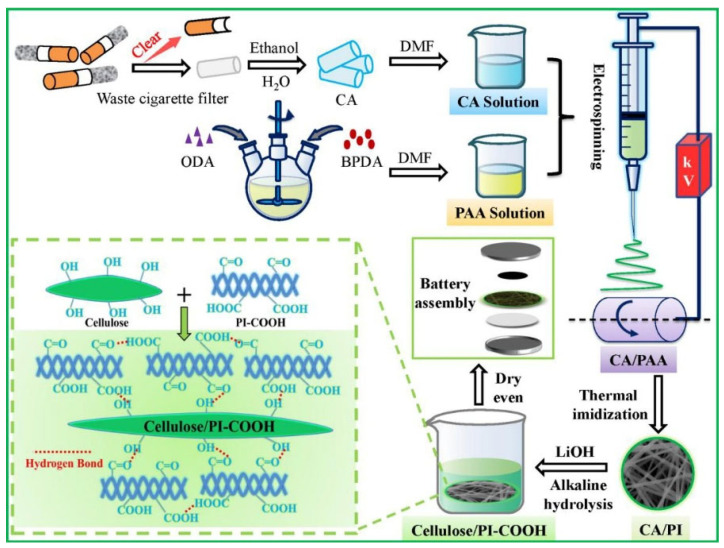
H-bond cross-linked cellulose/polyimide-COOH composite (Figure 3 reprinted/adapted with permission from Ref. [21]. 2022, Elsevier).

Surface crosslinking treatment can form a dense protective layer, significantly enhancing solvent resistance. Deng et al. [21] fabricated composite nanofiber membranes through electrospinning of cellulose acetate and PAA. Subsequently, alkaline hydrolysis of cellulose acetate exposed abundant hydroxyl groups on the surface, while partial imide ring opening of polyimide converted it into polyimide-COOH. These components formed a cross-linked structure through intermolecular hydrogen bonding at the molecular level, thereby constructing an interlocked 3D interconnected architecture. As illustrated in Figure 3, this unique structure endowed the material with enhanced electrochemical performance and improved safety characteristics.

#### 2.2.3. Friction Improvement

Conventional polyimides exhibit relatively high coefficients of friction (COF) and inadequate wear resistance, which can lead to surface abrasion and interfacial failure under dynamic contact or mechanical sliding conditions, ultimately compromising the reliability and service life of electronic devices. To enhance its tribological properties, researchers have conducted studies on improving the tribological performance of polyimide materials and achieved significant progress. Research on fluorinated PI films prepared using 4,4′-hexafluoroisopropylidene diphthalic anhydride (6FDA) [22,23] demonstrates that fluorinated groups can effectively reduce the surface free energy of the films. As the proportion of fluorinated monomers increases, the wear resistance of the films improves accordingly, with more stable friction coefficients and higher PV (pressure, velocity) values. In terms of main chain modification [24], copolymerization studies with pyromellitic dianhydride revealed that polyimide films with higher proportions of o-phenylenediamine demonstrate the most excellent tribological properties, maintaining good lubricity under high loads, while pyromellitic polyimide shows poor transfer film formation capability, leading to continuously increasing friction coefficients. Filler modification is also crucial for the tribological performance of polyimide composites, with filler types mainly including nanoparticles (such as nano-Al_2_O_3_, nano-TiO_2_, nano-SiO_2_, silicon nitride nanoparticles (nano-Si_3_N_4_), carbon nanotubes (CNTs), etc.) and layered materials (such as graphite, fluorinated graphite, etc.) [25,26,27]. For example, nano-SiO_2_ can significantly enhance the friction-reducing and anti-wear properties of polyimide.

**Figure 4:**
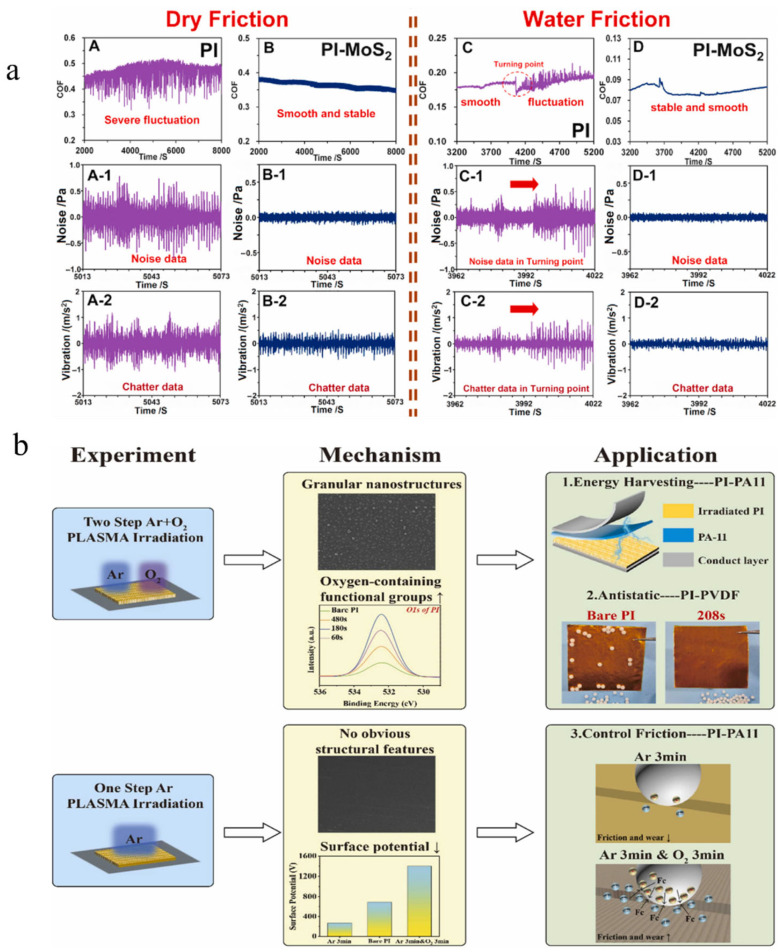
(**a**) Comparison of friction noise of polyimide and polyimide-MoS_2_ composite (Figure 4a reprinted/adapted with permission from Ref. [28]. 2021, Elsevier) (**b**) Enhancing friction resistance of polyimide through plasma surface treatment (Figure 4b reprinted/adapted with permission from Ref. [29]. 2022, Elsevier).

Liu et al. [28] developed a novel polyimide-based composite material by melt-mixing polyimide particles with MoS_2_ particles, followed by injection molding. This friction-optimized modification demonstrated outstanding performance, effectively reducing polyimide deformation on worn surfaces while significantly decreasing both frictional force and its fluctuation amplitude (Figure 4a). Additionally, plasma surface treatment can eliminate surface charges at friction interfaces, thereby regulating the coefficient of friction and improving wear resistance. As shown in Figure 4b, Sun et al. [29] employed an in situ two-step selective plasma treatment (Ar+O_2_) and a one-step Ar plasma treatment as surface modification methods. Following plasma irradiation, nanoparticle structures formed on the surface. With prolonged irradiation time, both the nanostructures and the quantity/coverage of oxygen-containing functional groups decreased. This demonstrates that plasma irradiation not only eliminates surface charges but also significantly enhances friction stability and wear resistance.

### 2.3. Fabrication of Porous Polyimide

Porous polyimide, as one of the most outstanding organic polymer foam materials, exhibits excellent mechanical properties, thermal stability, and thermal insulation characteristics. It can be fabricated into both flexible films and rigid substrates, finding wide applications in thermal insulation, noise reduction, fire protection, aerospace, building protection, electromagnetic shielding, dielectric substrates, electrical insulation, catalyst supports, and triboelectric materials [30,31,32,33,34,35]. In comparison with non-porous polyimide, porous polyimide offers several application advantages in the fields of electronic packaging and thermal management. The porous structure reduces the material density, making it more suitable for weight-sensitive electronic devices such as wearable devices and flexible electronic components. Meanwhile, the porous structure may enhance the material’s flexibility, enabling electronic packaging to remain stable in bending or vibrating environments. It can also lower the dielectric constant, which reduces signal transmission loss and improves the signal integrity of high-frequency electronic components. Additionally, it can buffer the thermal stress generated by electronic components during operation, reducing packaging cracking or component failure caused by thermal expansion and contraction. By controlling the size, distribution, and shape of the pores, the thermal conductivity of the polyimide can be flexibly adjusted to meet the personalized thermal management requirements of different electronic devices.

Generally, the porous structure relies on template preparation and removal methods, which can be classified into solid templates and liquid templates. Solid template methods typically use ice crystals, soluble salts, or polymer microspheres as sacrificial templates. In the freeze-drying process, a PAA solution is mixed with the template and rapidly frozen. The growth of ice crystals forms ordered pore channels, followed by freeze-drying to remove the ice template and thermal imidization to obtain ordered porous polyimide. The key to preparing porous polyimide via freeze-drying lies in controlling ice crystal formation during freezing. Studies show that different cooling sources (including low-temperature ethanol, liquid nitrogen, dry ice, and conventional refrigerators) lead to significantly different ice crystal growth kinetics [36,37]. These cooling sources exhibit notable differences in freezing rates and temperature gradients, directly affecting the pore structure characteristics of the final materials. Disordered freezing systems produce isotropic ice crystal networks when the precursor solution is immersed in a uniform freezing environment (e.g., low-temperature ethanol bath), resulting in disordered porous structures. Directional freezing systems achieve aligned ice crystal growth by establishing temperature gradients (e.g., placing molds above liquid nitrogen), ultimately yielding highly oriented tubular pore structures [38,39,40,41,42,43,44]. Such anisotropic structures demonstrate unique advantages in direction-dependent applications like thermal/electrical conduction.

**Figure 5:**
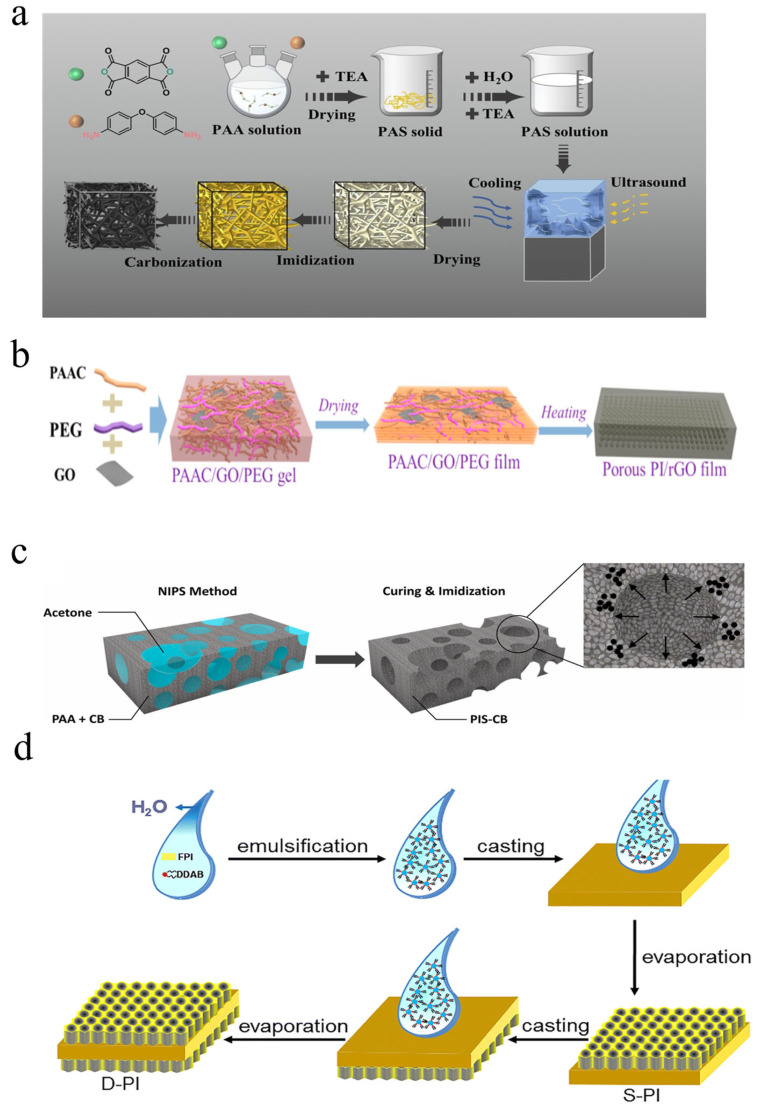
(**a**) Process flowchart for preparing porous polyimide via freeze-drying method (Figure 5a reprinted/adapted with permission from Ref. [45]. 2022, Elsevier). (**b**) Schematic diagram of porous polyimide fabrication using solid templating (Figure 5b reprinted/adapted with permission from Ref. [46]. 2019, American Chemical Society). (**c**) Nonsolvent-induced phase separation (NIPS) mechanism illustration (Figure 5c reprinted/adapted with permission from Ref. [47]. 2021, Elsevier). (**d**) Emulsion templating procedure for porous polyimide production (Figure 5d reprinted/adapted with permission from Ref. [48]. 2019, Springer Nature).

In addition to conventional ice-templating methods, the solid dispersant template approach provides an alternative, effective route for preparing porous polyimides. This method involves incorporating nanoparticles or thermally labile components as dispersed phases into the polymer matrix, followed by selective removal of these phases to construct porous structures. By introducing copolymers containing thermally labile blocks into polyimide precursors, pore formation and matrix curing can be achieved simultaneously during heating. As shown in Figure 5a, Xiao et al. [45] first introduced triethylamine into a PAA solution to form polyammonium salts through precipitation, followed by washing and drying processes. Subsequently, porous aerogels were obtained via freeze-drying and thermal imidization.

Commonly used thermally labile materials include poly (propylene oxide) [49], polyethylene glycol [46,50], and polycaprolactone [51], all of which decompose into volatile small molecules that escape upon heating. This process ingeniously combines the thermal degradation of porogens with the imidization reaction of polyimides, simplifying the fabrication procedure. As illustrated in Figure 5b, Zhao et al. [46] employed polyethylene glycol (PEG) as a pore-forming agent by blending it with polyamic acid carboxylate (PAAC) and graphene oxide (GO) in aqueous solution. The ternary PAAC/GO/PEG mixture formed a hydrogel through self-assembly, which was subsequently air-dried into xerogel films. Through in situ thermal decomposition of PEG during heat treatment, a porous thin-film structure was obtained. Notably, the homogeneous porous architecture could be precisely tuned by varying the mass ratio of PEG to PAAC/GO.

Furthermore, by controlling chemical reactions in the foaming slurry (e.g., generating gaseous products like CO_2_ or NH_3_), pores can be created within polyimide foams [52]. The uniqueness of this method lies in the concurrent completion of two processes during high-temperature curing: the imidization reaction of polyimide precursors and the formation of macro-porous foam structures through gas generation within the mold.

Liquid templating methods, as an important approach for preparing porous polyimide materials, primarily utilize liquid–liquid phase separation [53,54,55,56] or emulsion templating techniques [57,58,59,60,61,62] to construct controllable porous structures. Compared to solid templating methods, this approach offers superior structural tunability and process flexibility.

The nonsolvent-induced phase separation (NIPS) method prepares porous polyimides by controlling the phase separation behavior of the PAA-solvent-nonsolvent ternary system. Its core mechanism involves extracting the solvent (NMP/DMF) using a nonsolvent (e.g., water, ethanol, acetone) to form a two-phase structure with the polymer as the continuous phase and the poor solvent as the dispersed phase. After removing the poor solvent, thermal imidization yields porous polyimide. As depicted in Figure 5c, Kim et al. [47] fabricated porous composite sponges through a phase separation process between the solvent NMP and non-solvent acetone. During solvent evaporation, polymer solidification occurred with simultaneous formation of an internal porous structure. Following complete phase separation, the material was thermally cured in a high-temperature oven to achieve the final porous architecture. Li et al. [63] fabricated lightweight, high-performance EMI-shielding polyimide/reduced graphene oxide (rGO) composite foams by casting PAA/rGO and inducing phase separation with ethanol/distilled water as the nonsolvent. Similarly, Kim et al. [47] first dispersed carbon black (CB) fillers in PAA, then induced phase separation with acetone as the nonsolvent, and finally obtained polyimide/CB composite sponges through high-temperature curing and hot pressing. Yu et al. [64] successfully prepared thermally conductive aerogel fibers (PT-SCFs) with tunable pore-network and core-sheath structures by combining NIPS with coaxial wet-spinning.

Emulsion templating, as an efficient and controllable pore-forming technique, has been widely used in recent years for preparing porous polyimide materials. This method utilizes the self-assembly of oil-water interfaces in emulsion systems to form stable template structures, followed by thermal or chemical imidization to fix the pores, and finally removes the templating agent to obtain porous polyimide. As shown in Figure 5d, Xu et al. [48] developed porous polyimide films by utilizing didodecyldimethylammonium bromide (DDAB) and distilled water to create an emulsion template, which was subsequently evaporated to form the final porous membrane structure. Depending on the emulsion type, it can be classified into oil-in-water (O/W) [65,66], water-in-oil (W/O) [67,68], and high internal phase emulsion (HIPE) templating methods [69,70]. For example, Song et al. [66] used dodecane and lubricating oil as the oil phase with sodium dodecyl sulfate (SDS) as the surfactant to prepare three types of O/W emulsions (large dodecane droplets, small dodecane droplets, and small lubricating oil droplets), which were electrospun into fiber membranes demonstrating excellent separation performance and regeneration capability. Xu et al. [48] prepared emulsions by ultrasonically treating organic solvents in water, followed by filtration. The solution composed of micro-emulsion droplets was cast on polyimide substrates and transformed into sandwich-structured films through evaporation. The HIPE templating method forms three-dimensional interconnected pore channels through high-volume-fraction internal phases, significantly improving porosity. Cho et al. [68] developed a Pickering emulsion system (both O/W and W/O) that stabilizes without surface modification. Song et al. [62] used pyromellitic dianhydride-oxydianiline oligoimide particles synthesized in water and PAAS synthesized in NMP to prepare O/W Pickering HIPEs with internal phase ratios up to 85 vol%, followed by freeze-drying and imidization to prepare polyHIPEs with porosity up to 92%. Additionally, Park et al. [69] successfully synthesized porous polyimides with porosity >99 vol% using PAA salts as macromolecular surfactants and matrices.

## 3. Functional Polyimides and Composites

With the ever-increasing demands for material performance in packaging and thermal management of modern electronics with increasing power density and diverse capabilities, molecular structure design and composite engineering have emerged as research hotspots for endowing polyimides with novel functional properties.

### 3.1. Molecular Structure Design

As a class of high-performance polymers characterized by imide ring structures, polyimide possesses unique molecular designability that provides vast opportunities for precise performance modulation. Essentially formed through imidization reactions between various polymers containing specific linking segments, this structural characteristic enables targeted molecular engineering of polyimide structures to meet specific application requirements. From a molecular structure perspective, the thermophysical properties of polyimide are primarily determined by two key factors: (1) the chemical structures of the dianhydride and diamine monomers constituting the polymer backbone, (2) the overall molecular configuration formed through imide ring linkages. This well-defined structure-property relationship allows researchers to precisely control critical parameters such as dielectric constant and thermal properties through strategies including monomer selection, chain conformation regulation, and introduction of functional side groups. For instance, when polyimide films with extreme thermal stability are required, dianhydride and diamine monomers with rigid aromatic ring structures can be selected [71,72]. If targeting flexible electronic applications, introducing flexible segments or twisted non-coplanar structures can enhance material flexibility and film-forming properties [73,74,75].

#### 3.1.1. Low Dielectric Constant Polyimide

The dielectric constant of conventional polyimide films, which has a typical value of 3.2–3.4 [76], can no longer meet the requirements of 5G communication and advanced packaging technologies, driving researchers to seek breakthroughs at the molecular design level. Leveraging the strong molecular designability of polyimides, multi-level dielectric optimization strategies have been developed: introducing fluorine-containing groups or bulky side chains to reduce molecular polarizability, creating porous structures to increase free volume, employing special heterocyclic structures to regulate electron cloud distribution, and controlling molecular chain orientation to achieve dielectric anisotropy. These molecular engineering approaches not only significantly reduce the dielectric constant (achieving 1.4–2.5) [77] but also maintain the inherent excellent thermal stability and mechanical properties of polyimides.

**Figure 6:**
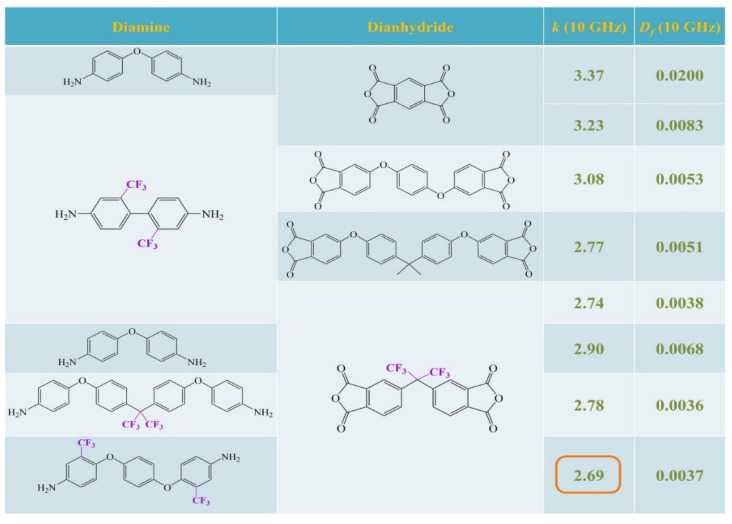
Effects of fluorinated group incorporation on the dielectric constant (*D_k_*) and dielectric loss (*D_f_*) of polyimide (Figure 6 reprinted/adapted with permission from Ref. [77]. 2022, Elsevier).

Fluorine atoms exhibit both low molecular polarizability and large free volume. The introduction of fluorine into polyimide can effectively reduce molecular polarizability, increase steric hindrance, and enhance molecular chain disorder, thereby lowering the dielectric constant of polyimide. Currently, polyimide fluorination is primarily achieved through two approaches: incorporation of fluorinated monomers [78,79,80,81] and direct fluorination reactions [82,83]. Wu et al. [77] synthesized seven fluorinated monomers for preparing fluorinated PIs. The results demonstrated that the introduction of fluorinated groups significantly improves dielectric properties. As shown in Figure 6, polyimide containing both fluorinated dianhydride and diamine monomers exhibited the most excellent dielectric performance (*D_k_* = 2.69, *D_f_* = 0.0037). This is mainly attributed to the low polarizability and strong electronegativity of fluorine atoms, which effectively restricts the conjugation effect of benzene rings. Similarly, Tao et al. [81] synthesized two types of fluorinated dianhydrides and four types of fluorinated diamine monomers. Their results indicated that increasing fluorine content leads to a linear decrease in the dielectric constant of polyimide, but this comes at the cost of reduced mechanical properties of the films, presenting a trade-off that requires careful consideration. Additionally, Park et al. [83] introduced fluorine into polyimide through fluorination reactions, demonstrating that the dielectric constant of polyimide decreases linearly with increasing fluorine pressure.

**Figure 7:**
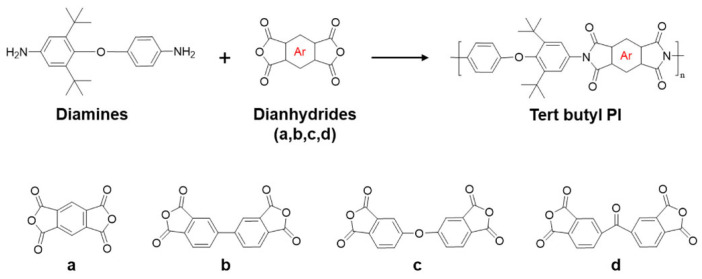
Synthesis of tert-butyl polyimide film with different dianhydries: (**a**) pyromellitic dianhydride, (**b**) biphenyl tetracarboxylic dianhydride, (**c**) 4,4’-oxydiphthalic anhydride, (**d**) 3,3’,4,4’-benzophenone tetracarboxylic dianhydride (Figure 7 reprinted/adapted with permission from Ref. [85]. 2024, MDPI).

Furthermore, the introduction of bulky side groups into the main chain can disrupt the original coplanar ordered structure, thereby reducing the dielectric constant. The incorporation of tert-butyl groups [84] as bulky side groups enhance the free volume of polyimide molecular chains and weakens intermolecular interactions, leading to decreased dielectric constant and loss. Li et al. [85] utilized adamantane as a bulky side group and PEG as a porogen to prepare porous membranes (Figure 7). The combined effects of bulky side groups and porous structures reduced the polyimide dielectric constant to 1.85. Briefly, since air has a dielectric constant of 1, introducing nanoscale micropores into polyimide can significantly lower the dielectric constant. Kim et al. [86] fabricated porous polyimide membranes via phase separation, achieving a dielectric constant as low as 1.32 (see Section 2.3 for details on porous polyimides). Additionally, incorporating fillers such as polyhedral oligomeric silsesquioxane (POSS), fluorinated graphene, or polytetrafluoroethylene (PTFE) [87,88] can effectively reduce polarization per unit volume, thereby lowering the dielectric constant.

#### 3.1.2. Thermal Stable Polyimide

**Figure 8:**
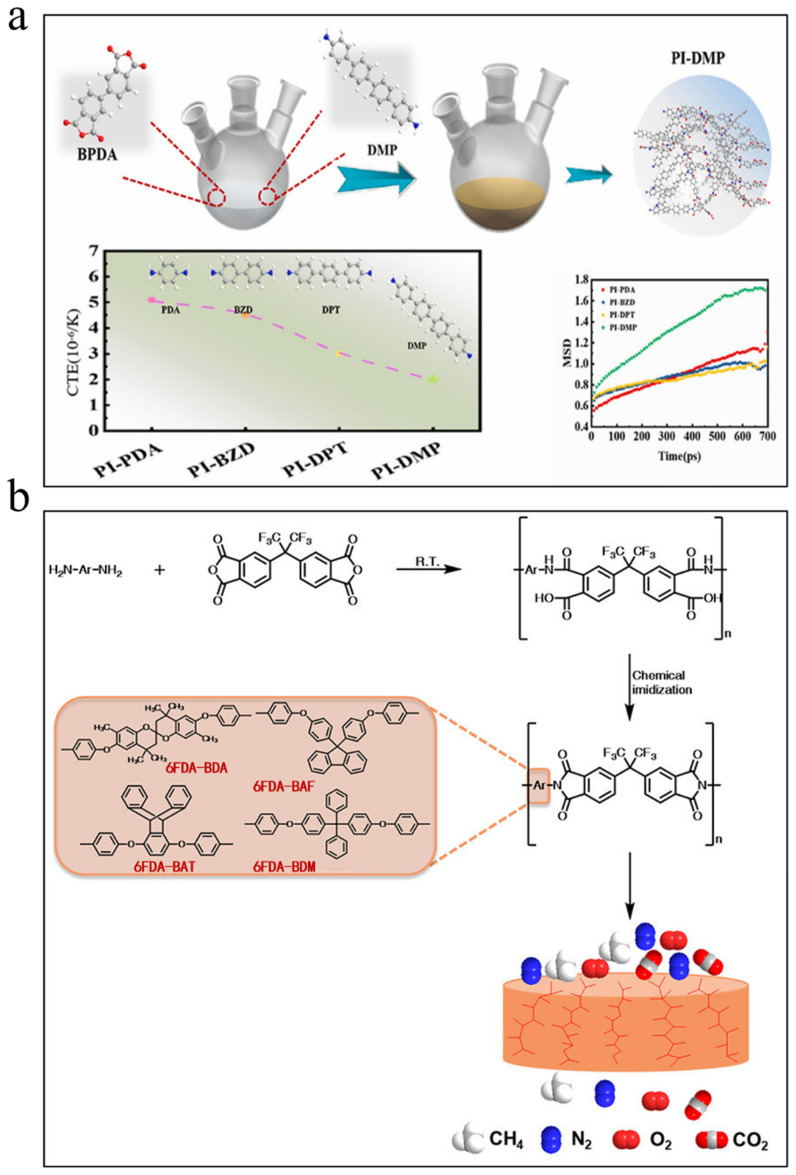
(**a**) Aromatic rigid segments introduced in the polymer backbone to enhance thermal stability of polyimide (Figure 8a reprinted/adapted with permission from Ref. [93]. 2024, Elsevier). (**b**) Non-coplanar rigid diamine monomer polymerized with 6FDA to prepare polyimide with improved thermal stability (Figure 8b reprinted/adapted with permission from Ref. [71]. 2022, American Chemical Society).

The ongoing miniaturization, integration, and high-frequency development of electronic devices have exposed the limitations of conventional polyimides under extreme thermal conditions (e.g., high-temperature reflow soldering and prolonged thermal aging). These materials face critical challenges in performance stability, manifesting as thermal decomposition, dimensional distortion, and interfacial delamination-all of which significantly compromise packaging reliability. Consequently, enhancing thermal stability has become paramount for ensuring reliable encapsulation in advanced electronic systems. The optimization of thermal stability in polyimide requires systematic consideration across multiple dimensions, including chemical bond energy, molecular chain rigidity, and intermolecular interactions. Studies demonstrate that introducing rigid aromatic structures (e.g., biphenyl [89,90], naphthalene rings [91,92]) into the polymer backbone significantly enhances the glass transition temperature (T_g_), as these rigid configurations effectively restrict molecular chain segment mobility. For instance, Li et al. [93] synthesized polyimide films using four benzene-structured diamines and found that increasing the number of benzene rings while reducing imide rings in the horizontal structure improved the coefficient of thermal expansion (CTE), dielectric properties, and thermal stability (Figure 8a). Concurrently, incorporating monomers with high-bond-energy chemical linkages (e.g., C-F bonds [94,95,96]) substantially elevates the thermal decomposition temperature (T_d_). Notably, regulating intermolecular interactions is equally critical: introducing secondary interactions like hydrogen bonding [97] enhances thermal stability without compromising processability. Novel structural units such as adamantane [98] offer innovative pathways for thermal stability enhancement, where bulky groups not only increase chain rigidity but also suppress high-temperature molecular motion via steric hindrance. However, the improvement in thermal stability is often accompanied by a decline in processability, which necessitates a balance through molecular design: on one hand, introducing an appropriate amount of flexible chain segments [99] can enhance solubility, while on the other hand, adopting asymmetric or twisted non-coplanar [71] structures can disrupt the regular arrangement of molecular chains (Figure 8b).

#### 3.1.3. Structure–Property Relationship of Polyimide

At the molecular level, the aromatic rings and imide rings in the main chain form strong intermolecular interactions through π-π stacking and charge transfer complexes, significantly enhancing thermal stability and mechanical strength [100]. Meanwhile, the coplanarity and conjugation effects of aromatic rings optimize chemical resistance and dielectric properties. A clear structure–property relationship enables precise material performance regulation through strategies including monomer selection, molecular chain conformation control, and functional side group incorporation.

Specifically, the introduction of fluorine-containing side groups reconstructs electron cloud distribution through their strong electronegativity. The dipole moment of C-F bonds and the inductive effect generated with the π-electron system of the main chain significantly reduce electron delocalization, thereby lowering the dielectric constant. However, the large van der Waals radius of fluorine atoms simultaneously weakens intermolecular interactions, leading to reduced mechanical strength [78]. For instance, Zhao et al. [101] introduced trifluoromethyl groups to decrease the dielectric constant from 3.78 to 3.21, but this also reduced tensile strength from 127 MPa to 77 MPa. Bulky side groups (e.g., tert-butyl) disrupt the regular arrangement of molecular chains through steric hindrance effects, increasing free volume to reduce dielectric constant, though such structural disorder compromises crystallinity and mechanical properties. The performance influence of porous structures essentially stems from quantum confinement effects at dielectric interfaces: when pore sizes are below 100 nm, localized electronic states form at gas–solid interfaces, and this interfacial polarization effect effectively reduces dielectric constants.

Regarding thermal stability, incorporating rigid aromatic structures (e.g., biphenyl, naphthalene rings) can significantly raise the glass transition temperature, as these rigid configurations effectively restrict molecular chain segment mobility. However, such constrained molecular motion weakens energy dissipation mechanisms during crack propagation, resulting in increased brittleness. Notably, introducing moderately distorted non-coplanar structures maintains high rotational energy barriers while providing additional energy dissipation pathways through “local bending” of molecular chains—precisely the molecular mechanism enabling simultaneous high thermal stability and toughness. For example, Shi et al. [102] incorporated dianhydride with ether linkages; at an ether-linked to fluorinated dianhydride ratio of 4:6, the tensile strength reached 135.3 MPa and elongation at break 8.3%, representing 109.6% and 118.45% improvements, respectively, over the pristine film without ether linkages.

These multiscale structure–property relationships provide clear guidance for designing next-generation polyimide materials for advanced packaging and thermal management applications, where simultaneous optimization of dielectric, thermal, and mechanical properties is critical.

### 3.2. Composite Engineering

As a stable substrate material, polyimide can be compounded with other fillers to enhance its intrinsic properties or impart additional functionalities. The key to performance enhancement lies in the synergistic mechanisms of interaction between fillers and the polyimide matrix.

#### 3.2.1. Thermal Conductive Polyimide Composites

In solid materials, heat conduction is mediated by thermal carriers such as electrons, phonons, and photons. The thermal conductivity (*k*) of solid materials can be described by *k* =$\;\frac{1}{3}$*C*_p_*vl*, where *C*_p_ represents the material’s specific heat capacity at constant pressure; *v* denotes the average velocity of thermal carriers; and *l* corresponds to the mean free path of thermal carriers. In polyimides, phonons serve as the primary carriers for heat transfer, and the thermal conductivity strongly depends on the phonon mean free path and transportation velocity. The transport behavior of phonons is significantly influenced by phonon scattering phenomena. The disordered molecular chains within polyimides cause phonons to collide frequently with chain segments and defects, strongly shortening the mean free path. Moreover, the weak interchain interaction through van der Waals forces limits heat transfer between chains. Additionally, most polyimides have an amorphous or low-crystallinity structure, lacking a regular lattice arrangement to facilitate phonon transportation. Voids, bubbles, or impurities that can be introduced during the synthesis processes can also serve as scattering centers to limit heat transfer. These factors severely impede thermal diffusion via phonons within the polyimides [103].

To enhance the effective thermal conductivity, various types of fillers with higher thermal conductivity, including zero-dimensional particles, one-dimensional nanotubes and nanowires, two-dimensional nanosheets, and three-dimensional skeletons, have been compounded with polyimide. Based on the simple mixture rule, the thermal conductivity of the resultant polyimide can be expected to linearly increase with increasing loading of fillers. Boosted thermal conductivity typically occurs when the added fillers form percolated thermal networks within the polyimide matrix.

**Figure 9:**
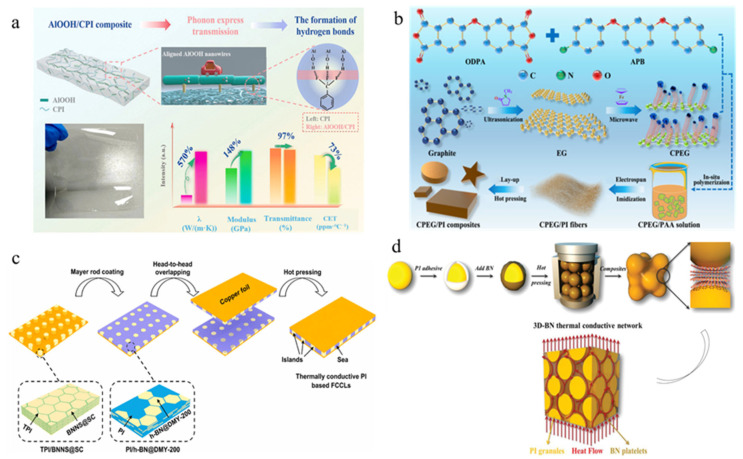
(**a**) Enhancing thermal conductivity of polyimide through compounding with one-dimensional AlOOH nanowire fillers (Figure 9a reprinted/adapted with permission from Ref. [113]. 2024, Springer Nature) (**b**) Increasing thermal conductivity of polyimide through adding expanded graphite/CNT hybrid fillers (Figure 9b reprinted/adapted with permission from Ref. [112]. 2021, Springer Nature). (**c**) Increasing thermal conductivity of polyimide through loading two-dimensional hexagonal boron nitride (h-BN) fillers (Figure 9c reprinted/adapted with permission from Ref. [119]. 2022, Elsevier). (**d**) Hot-pressed 3DBN network in polyimide matrix (Figure 9d reprinted/adapted with permission from Ref. [121]. 2019, Wiley).

The intrinsic properties of high-thermal-conductivity fillers and preparation methods have a profound influence on the thermal performance of composites. Metallic fillers (e.g., silver, copper) [104,105,106] can make use of abundant free electrons for efficient heat conduction. Guo et al. [107] employed glucose to simultaneously reduce Ag^+^ and GO in solution, preparing silver nanoparticle-decorated reduced graphene oxide (Ag/rGO), which was then hot-pressed into Ag/rGO/polyimide nanocomposites, achieving a thermal conductivity of 2.12 W/(m·K). In comparison with spherical particles, it is easier for one-dimensional fillers [108,109,110,111,112] (carbon nanotubes and metal nanowires) to form connected thermal pathways due to their high aspect ratios. Ma et al. [113] introduced AlOOH nanowires into polyimide to prepare composite films (Figure 9a). The in-plane alignment of AlOOH nanowires facilitated the formation of thermal conduction paths, resulting in composite films with in-plane thermal conductivity more than five times higher than pure polyimide, reaching up to 3.568 W/(m·K). Similarly, Guo et al. [112] employed a microwave-assisted method to grow vertically aligned carbon nanotubes on exfoliated graphite as thermal conductive fillers in polyimide composite films. The resultant composite films not only exhibited significantly higher thermal conductivity than pure polyimide but also maintained nearly unchanged thermal conductivity after 1000 thermal cycles (Figure 9b).

2-D fillers (graphene, boron nitride nanosheets) [114,115,116,117,118] have also received intensive research attention to enhance the thermal conductivity of polyimide due to their efficient in-plane phonon transport and high aspect ratios. Lan et al. [112] developed polyimide composites loaded with maleimide-modified graphene nanosheets (M@GNS), where the multi-faceted covalent bonds and oriented graphene alignment created through covalent bonding and coating processes established low-thermal-resistance interface structures, achieving an exceptionally high in-plane thermal conductivity of 16.1 W/(m·K). Xiao et al. [119] incorporated micron-sized hexagonal boron nitride (h-BN) into polyimide, demonstrating a 13-fold enhancement in in-plane thermal conductivity and nearly 3-fold improvement in through-plane conductivity (Figure 9c). Notably, when the BN loading was increased by just 1%, both in-plane and through-plane thermal conductivities were further doubled.

Three-dimensional fillers such as expanded graphite and hybrid 3D materials [120,121,122] offer continuous network structures to establish thermal pathways. Zhang et al. [120] constructed an interpenetrating carbon nanotube (CNT) network in polyimide/boron nitride nanosheet (polyimide/BNNS) films, where CNTs served as bridge fillers to create a dual thermal conductive network. Remarkably, with only 0.3 wt% CNT loading, the composite film achieved a 100% improvement in thermal conductivity. As shown in Figure 9d, Ding et al. [121] employed a polyimide adhesive to coat BN platelets onto polyimide particle surfaces. Through hot-pressing, the well-aligned and tightly connected BN platelets formed an effective 3D thermal network within the polyimide matrix. The polyimide/BN composite exhibited an outstanding thermal conductivity of 4.47 W/(m·K) at a low BN loading of 20 vol%, representing 2099% enhancement over pure polyimide.

#### 3.2.2. Mechanical Robust Polyimide Composites

The mechanical properties of polyimide are excellent, and its performance is closely related to molecular structure, degree of polymerization, processing technology, and whether fillers are added. The tensile strength of polyimide is typically within the range of 100–150 MPa, some high-performance polyimides can reach over 170 MPa. The elongation at the break of polyimide is generally low (5–15%), showing brittle characteristics. Through introducing flexible chain segments; however, it can be increased to over 50%. The typical bending strength and bending modulus of rigid polyimide are approximately 150–200 MPa and 2–4 GPa. Compounding polyimides with other fillers can significantly enhance their mechanical properties.

**Figure 10:**
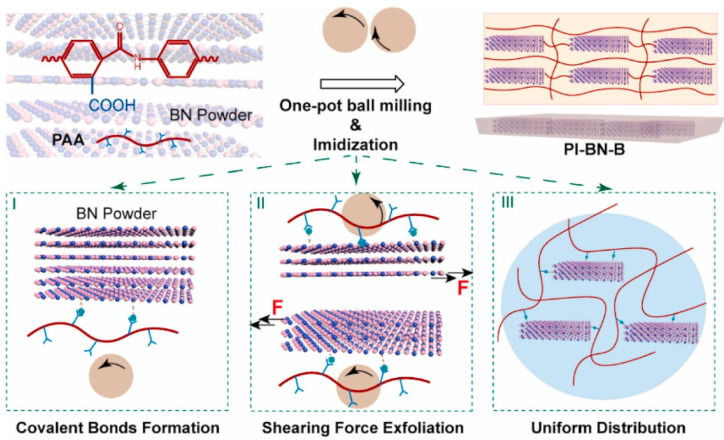
Mechanochemical-assisted fabrication of polyimide-BN-B composite films to enhance mechanical properties. (Figure 10 reprinted/adapted with permission from Ref. [123]. 2021, Elsevier).

The mechanical properties of polyimide are excellent, and its performance is closely related to molecular structure, degree of polymerization, processing technology, and whether fillers are added. One-dimensional fibrous fillers (e.g., CNTs, metal nanowires) can inhibit crack propagation via bridging effects, while two-dimensional platelet fillers (e.g., BN) enhance toughness through interfacial sliding mechanisms. Reduced graphene oxide (rGO) forms strong interfacial bonding with the polyimide matrix through its oxygen-rich surface functional groups. Three-dimensional network fillers construct stereoscopic reinforcement frameworks—for instance, AlOOH nanowires [113] incorporated into polyimide films increased Young’s modulus by 148% and reduced the coefficient of thermal expansion (CTE) by 27% while maintaining excellent flexibility. As shown in Figure 10, Ou et al. [123] reported that the addition of BN (20%) could decrease the CTE of polyimide by 41%, and the addition of 10% BN could improve the tensile strength by 113%. Remarkably, ionic liquid-grafted BN prepared by ball milling achieved 93.62% tensile strength enhancement at just 4 wt% loading [124].

Quantum dot doping technology provides an innovative solution for enhancing the mechanical properties of polyimide composites. Das et al. [125] utilized heteroatom-doped carbon dots to reinforce polymer films, simultaneously improving their flexibility, oxidation resistance, and UV stability. Suleiman et al. [126] successfully integrated MXene quantum dots into thermoplastic chitosan via a wet-chemical mixing approach, fabricating composite films with outstanding UV-blocking capability (>90%), remarkable antioxidant activity (>78%), and excellent uniaxial tensile strength of 4–5 MPa.

#### 3.2.3. EMI-Shielding Polyimide Composites

When electromagnetic waves encounter shielding materials, their attenuation typically occurs through three primary mechanisms: (1) reflection loss (SER) at the material surface, (2) absorption loss (SEA) through energy conversion to heat within the material, and (3) multiple-reflection loss (SEM) from interfacial reflections inside the shielding structure. Therefore, the total electromagnetic shielding effectiveness (SET) is the sum of these three components, i.e., SE_T_(dB) = SE_R_ + SE_A_ + SE_M_. The EMI shielding performance can be characterized by EMI SE, which is defined as the decibel-scaled ratio of incident power to transmitted power through the shielding material. Materials with EMI SE between 10 and 30 dB generally provide insufficient electromagnetic shielding, while those exceeding 30 dB can attenuate up to 99.9% of incident radiation [127].

The enhancement of electromagnetic interference (EMI) shielding performance in polyimide composites primarily depends on the morphology and type of conductive fillers. Currently, the most commonly used conductive fillers include metals, carbon-based materials, and hybrid conductive composites. Metallic fillers primarily achieve high shielding effectiveness through free-electron reflection, while carbon-based materials enhance electromagnetic wave absorption via dielectric polarization and multiple reflections. Additionally, interfacial interactions between the fillers and polyimide matrix generate additional interfacial polarization loss, further optimizing the overall shielding performance.

Metallic fillers (e.g., silver and copper) [128,129,130,131] rely on free electron reflection to rapidly establish highly conductive networks. For instance, three types of silver nanostructures—silver nanospheres (AgNSs), silver nanowires (AgNWs), and silver nanowire-nanoplate hybrids (AgNWPs)—were compared. Results showed that AgNWPs formed a denser 3D conductive network at the same filler loading, where the seamless interconnected AgNWP network provided rapid electron transport pathways within the foam, leading to superior EMI shielding performance in AgNWP/polyimide composites [132].

Carbon-based fillers (e.g., CNTs and graphene) [133,134,135,136,137] leverage their lightweight and high conductivity to form 3D conductive networks, achieving exceptional shielding effectiveness. Xiong et al. [134] employed a co-carbonization method to construct an integrated graphene/carbon fiber network, endowing polymer composites with simultaneously enhanced EMI shielding and thermal conductivity. Furthermore, the incorporation of MXenes combines their superior EMI shielding capability with polyimide’s stability, enabling cost-effective shielding networks. Cheng et al. [138] engineered a hierarchically porous polyimide/Ti_3_C_2_T_x_ (MXene) composite film. Remarkably, this ultrathin film (90 μm) delivered an outstanding absolute EMI shielding effectiveness of 15,527 dB·cm^2^/g, setting a new benchmark for lightweight shielding materials.

Polyimide composites achieve synergistic EMI enhancement through multicomponent systems like rGO/CNT/polyimide [139] and Ti_3_C_2_T_x_/ANF/PI [140]. Zhang et al. [139] fabricated porous polyimide composites using polyaniline-modified graphene oxide (PANI-g-GO) and multi-walled carbon nanotubes (MWCNTs) as hybrid fillers through an in situ polymerization process combined with thermal-assisted nonsolvent-induced phase separation. It is revealed that the molecular-level compatibility between PANI and polyimide chains significantly improved the dispersion of PANI-g-GO within the polyimide matrix, and the synergistic effect of well-dispersed PANI-g-GO and MWCNTs facilitated the formation of an efficient 3D conductive network in the porous polyimide composite.

#### 3.2.4. Comparison of Property Enhancement by Different Fillers

As shown in Table 2, the incorporation of various fillers at relatively low loadings can simultaneously enhance both the thermal conductivity and EMI shielding effectiveness of polyimide composites. Significant differences exist in the enhancement effects among different fillers.

Metallic fillers and carbon-based materials exhibit notable differences in thermal conductivity performance, primarily due to their distinct heat conduction mechanisms. Metallic materials achieve efficient heat transfer mainly through the movement of free electrons, with their long electron mean free paths contributing to extremely high intrinsic thermal conductivity. In contrast, heat conduction in carbon materials relies predominantly on phonon transport, which is susceptible to lattice defects and interface scattering. Particularly noteworthy is that o1-D CNTs, with their exceptionally high axial thermal conductivity and high aspect ratio advantages, can form thermal networks at relatively low filler loadings. While two-dimensional graphene demonstrates excellent in-plane thermal conductivity, the weak van der Waals forces between layers limit the actual thermal enhancement effect in composites.

The differences in EMI shielding effectiveness mainly stem from the various electromagnetic loss mechanisms of the materials. Metallic fillers primarily generate significant reflection loss through conductive networks formed by free electrons, a mechanism particularly effective in high-frequency ranges. Carbon materials, on the other hand, exhibit more balanced loss mechanisms, including dielectric loss (dipole polarization and interface polarization) and moderate conductive loss. Notably, two-dimensional transition metal carbides like MXene, with their unique surface terminations and layered structures, can simultaneously provide considerable dielectric loss and conductive loss, demonstrating superior comprehensive shielding performance. Additionally, the introduction of magnetic fillers (e.g., BaFe_12_O_19_) can further enhance magnetic loss, enabling broader frequency band electromagnetic wave absorption.

In general, thermophysical properties of polyimide composites are influenced by the loading of fillers, filler distribution, and compounding strategies. Although high loading is normally beneficial for enhancing thermal conductivity and EMI shielding performances, the original mechanical flexibility and processability of polyimide might be sacrificed. Most frequently, the formation of connected networks with minimum loading is desired. The interface characteristics between fillers and the matrix often play a crucial role in determining the final performance. For thermal conductivity, good interfacial bonding can reduce phonon scattering and lower interfacial thermal resistance. For EMI shielding, appropriate interface impedance matching can minimize electromagnetic wave reflection while enhancing absorption loss. Multi-component filler systems exhibit remarkable synergistic effects. Metal–carbon hybrid systems (e.g., Ag/rGO) combine the high conductivity of metals with the lightweight properties of carbon materials, while carbon-ceramic composite systems (e.g., GO/BN) achieve both high thermal conductivity and excellent insulation properties. These synergistic effects are not merely additive but may create new interfacial effects and coupling mechanisms. For instance, in MXene/CNT composite systems, the two-dimensional planar structure of MXene and the one-dimensional linear structure of CNTs can bridge each other, forming more complete thermal and conductive networks.

## 4. Electronic Packaging and Thermal Management Application

### 4.1. Flexible Electronic Packaging

**Figure 11:**
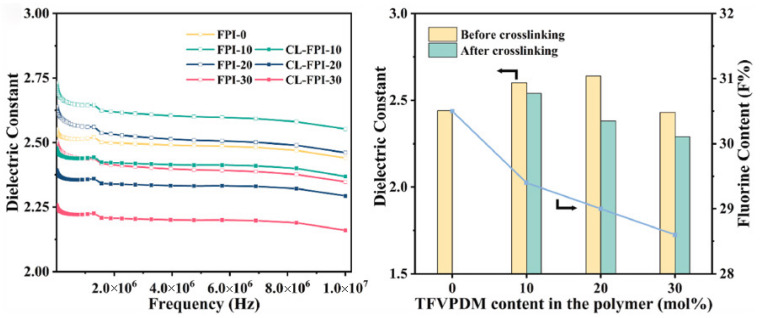
Polyimide with low dielectric constant for electronic packaging (Figure 11 reprinted/adapted with permission from Ref. [148]. 2023, Wiley).

With the rapid development of flexible electronic packaging technologies, comprehensive performance requirements have been raised for packaging materials, including low dielectric properties (*D_k_* < 3.2, *D_f_* < 0.005) [144,145] and matched coefficient of thermal expansion (CTE: 2–17 ppm/K) with substrates [146]. As a representative high-performance engineering plastic, polyimide demonstrates irreplaceable value in electronic packaging applications due to its unique molecular design flexibility and exceptional integrated performance characteristics. In 5G/6G communication and flexible electronics, where dielectric performance requirements are particularly stringent, polyimide achieves reduced dielectric constants (*D_k_*) through the introduction of air voids. Given air’s ultralow dielectric constant (*ε*_r_ ≈ 1), these porous structures effectively decrease the overall *D_k_* value according to the rule of mixture in composite materials [147]. The introduction of fluorinated groups and bulky side chains further reduces dielectric constants through synergistic effects, effectively addressing high-frequency signal loss challenges. As shown in Figure 11, Fan et al. [148] achieved a breakthrough by synergistically combining biphenyl/m-phenylene rigid skeletons with tetrafluorostyrene crosslinking networks, successfully reducing the dielectric constant of crosslinked fluorinated polyimides from 2.46–2.60 (existing FPIs) to 2.22–2.46. Concurrently, CTE matching is critical for packaging reliability. Mismatched CTEs between substrates and packaging materials may cause delamination or failure. Biphenyl-type PI films achieve ultra-low CTE (2 ppm/K) by increasing benzene rings and reducing imide rings, closely matching single-crystal silicon (3.6 ppm/K) [93]. These combined advantages establish PI as the core material for flexible circuit substrates and advanced electronic packaging.

### 4.2. EMI Shielding of Electronics

Emerging technologies like artificial intelligence and new energy vehicles are driving semiconductor components toward higher integration and operating frequencies [149,150]. The resulting complex EMI in confined spaces can severely disrupt electronic components, compromising device reliability and lifespan, with potential human health implications [151,152,153].

EMI shielding mechanisms include absorption, reflection, and attenuation. Polyimide composites incorporating conductive materials—including the aforementioned silver nanospheres (AgNSs), silver nanowires (AgNWs), silver nanoplates (AgNPs), MWCNTs, MXenes, and graphene—have been extensively studied for EMI shielding applications. These hybrid systems leverage the unique electrical and structural properties of each filler to construct high-performance shielding architectures through: (1) free-electron-dominated reflection in metallic networks; (2) synergistic absorption-conduction in carbon frameworks; (3) ultrahigh interfacial polarization loss in MXene heterostructures [132,134,138,154]. Liu et al. [155] physically sprayed silver coatings, endowing polyimide with electrical conductivity and EMI shielding ability. In the frequency range of 200–7000 MHz, the EMI shielding efficiency of silver plating on one surface and two surfaces ranged from 36.4 to 60.7 dB and 61.6–95.6 dB, respectively. The results showed that silver plating greatly improved the electromagnetic shielding ability of polyimide. Building on this foundation, researchers have explored incorporating conductive materials during polyimide synthesis to achieve more uniform EMI shielding. Studies demonstrate that constructing continuous 3D conductive networks within the polymer matrix represents one of the most effective strategies for enhancing EMI shielding capabilities. Cheng et al. [156] reported a graphene-polyaniline (Gr-PANI) hybrid filler system and fabricated flexible polyimide composite films with exceptional EMI shielding performance via in situ polymerization. The composites achieved an ultrahigh specific shielding effectiveness of 4096.2 dB·cm^2^·g^−1^.

**Figure 12:**
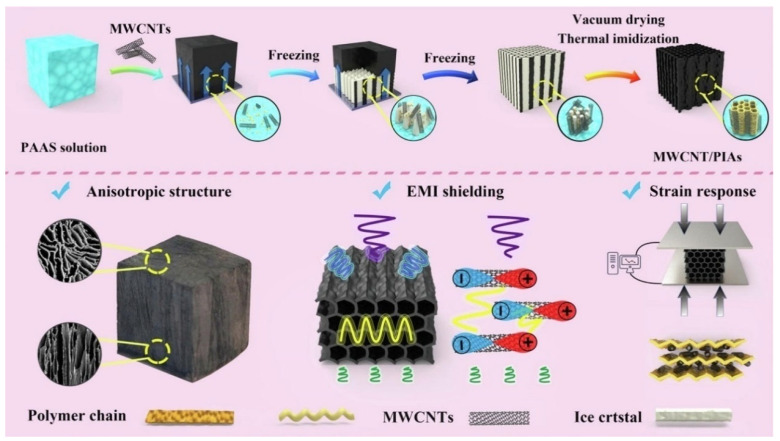
Polyimide composites for high-performance EMI shielding (Figure 12 reprinted/adapted with permission from Ref. [158]. 2022, Elsevier).

Furthermore, the porous structure of polyimide not only serves as a substrate for loading fillers but also enhances EMI shielding through electromagnetic wave absorption. As demonstrated by Yu et al. [157] in polyimide/graphene anisotropic aerogels, the EMI shielding effectiveness was lower along the pore direction compared to the direction perpendicular to the pores, likely due to the difficulty of electromagnetic waves penetrating through pore walls. Ma et al. [158] fabricated a series of multifunctional aerogels composed of PI and MWCNTs through unidirectional freeze-casting, vacuum freeze-drying, and thermal imidization processes. These aerogels exhibited both flexibility and robustness in both vertical and parallel directions, while demonstrating anisotropic electromagnetic interference shielding effectiveness (EMI SE), with values decreasing from 99.4 dB to 46.9 dB between the two orientations (Figure 12).

### 4.3. Thermal Management Application

With the continuous advancement of electronic device miniaturization and high-performance demands, thermal management has emerged as a critical challenge. Across increasingly integrated microelectronics, communication equipment, aerospace systems, and new energy vehicles, effective heat dissipation to prevent performance degradation or system failures caused by overheating has become a decisive factor for sustainable technological development. In diverse thermal management applications, polyimide demonstrates dual functionality for both thermal insulation and thermal conduction.

#### 4.3.1. Thermal Insulation

Localized heat accumulation and thermal crosstalk have become primary causes of failure in electronic packaging systems. For high-density integrated chips, where power density has increased by an order of magnitude, these thermal issues can induce significant system performance fluctuations. The thermal resistance and stability of aromatic moieties in polyimide molecular chains, combined with porous structures, enable ultra-low thermal conductivity. In porous structures, the interconnected 3D network creates tortuous heat transfer pathways for polymer molecules, inducing diffusive thermal transport that effectively reduces solid-phase thermal conductivity. Simultaneously, the increased solid–gas interfaces generate multi-reflection effects that further suppress heat conduction. As reported by Zhao et al. [159], high-porosity polyimide aerogels demonstrate superior thermal insulation performance compared to commercial materials, which was visually evidenced by the infrared thermal imaging when they were placed on heating platforms. Qian et al. [160] fabricated polyimide nanofiber aerogels (PINFAs) that demonstrated remarkable thermal stability, showing only 0.7% volume shrinkage and 3.5% mass loss after 240 h of heating at 300 °C in air. Even after this extreme thermal exposure, the PINFAs maintained 95% compressibility with complete shape recovery upon unloading, conclusively validating their exceptional thermal and mechanical performance. These properties significantly outperform conventional commercial foam materials, which typically exhibit 5–8% volume shrinkage and 10–15% mass loss under identical conditions while failing to recover more than 60% of their original shape.

**Figure 13:**
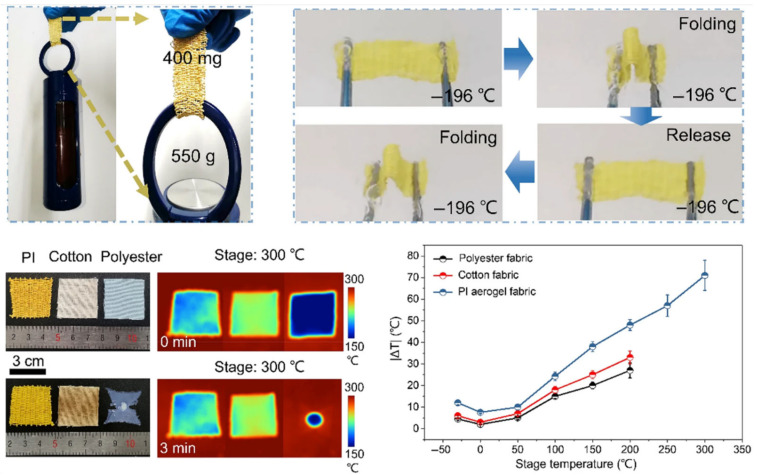
Polyimide aerogel for thermal insulation applications (Figure 13 reprinted/adapted with permission from Ref. [161]. 2022, Springer Nature).

Furthermore, Wang et al. [18] developed a biomimetic strategy inspired by polar bear hair, wherein an aqueous PAA gel was employed as the spinning precursor. Subsequent freeze-drying preserved the hierarchical porous architecture, followed by thermal imidization to yield polyimide fibers. The resultant woven textiles demonstrated exceptional thermal insulation properties coupled with self-extinguishing behavior under elevated temperatures, rendering them promising candidates for thermal insulation, energy conservation, and protective applications. In a parallel study, Xue and coworkers [161] fabricated lightweight and super-insulating polyimide aerogel fibers via cryogenic spinning, utilizing polyvinyl alcohol (PVA) as a pore-forming modulator. The as-synthesized polyimide aerogel fibers exhibited a high porosity of 95.6% with uniformly distributed submicron pores, while maintaining remarkable mechanical robustness and flexibility. Such characteristics enabled their direct integration into textile architectures for personalized thermal management applications (Figure 13).

#### 4.3.2. Thermal Conduction

**Figure 14:**
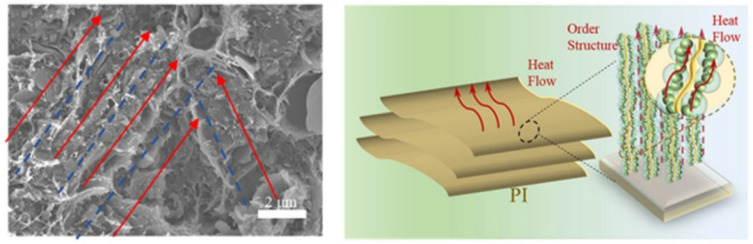
Polyimide composite film with high thermal conductivity for thermal management applications. The red arrows indicate the orientation of fillers and the direction along which heat transfer is enhanced. (Figure 14 reprinted/adapted with permission from Ref. [164]. 2023, Wiley).

In the field of microelectronics, contrary to thermal insulation applications, enhancing thermal conductivity is critical to prevent localized heat accumulation that may cause damage to electronic components. Currently, the average heat flux in chips ranges from 10 to 50 W/cm^2^, but this value can increase more than fivefold in hotspot regions [162]. Against this backdrop, the thermal conductivity design of polyimide-based thermal management materials has become a central research focus in this field. To improve the thermal conductivity of polyimides, researchers have reported the construction of additional heat diffusion pathways within the polyimide matrix. The introduction of highly thermally conductive fillers, such as metals, BN, and graphene, can significantly enhance the composite’s thermal conductivity. Herein, we highlight some other research efforts beyond those described in Section 3.2. Guo et al. [122] developed a hierarchically structured multifunctional composite film, wherein the top layer, composed of graphene oxide/expanded graphite (GO/EG) was fabricated via filtration and hot-pressing methods to provide dual functionality of thermal conduction and EMI shielding. The base layer consisted of electrospun polyimide fibers designed to enhance mechanical robustness. These two layers were integrated through in situ polymerization and doctor-blade coating of Fe_3_O_4_/polyimide interlayer, which synergistically improved EMI shielding performance. The resultant composite film exhibited exceptional in-plane thermal conductivity of 95.4 W/(m·K), an outstanding EMI shielding effectiveness of 34.0 dB, considerable tensile strength (93.6 MPa), and rapid electrothermal response (5 s). Dong et al. [163] fabricated a trilayer porous polyimide composite film (denoted as PSLS) through a combination of hot-pressing and liquid crystal-induced phase self-assembly, comprising a symmetric structure of surface CaF_2_-PI layers (sCaF_2_-PI) and a central CaF_2_ layer (LCaF_2_). The as-prepared PSLS film demonstrated an ultralow dielectric constant of 1.89 coupled with an exceptionally high in-plane thermal conductivity of 13.58 W/(m·K), while maintaining superior electrical insulation properties. As shown in Figure 14, Song et al. [164] developed polyimide composites with ordered structures by bridging boron nitride-filled commercial polyimide with aramid nanofibers. At 30 wt% loading, the thermal conductivity increased to 1.162 W/(m·K), representing an eight-fold enhancement over pristine polyimide.

Furthermore, the synergistic effect between porous polyimide and phase change materials (PCMs) enables dynamic thermal regulation under variable temperatures. Shi et al. [165] developed a novel flexible and foldable composite film based on polyimide/phosphorene (PR) hybrid aerogel and PEG, achieving an ultrahigh PEG loading capacity exceeding 90 wt% with a phase change enthalpy over 150 J/g. This composite demonstrated effective thermal camouflage performance lasting for more than 12 min in target environments. Cao [166] fabricated MXene/polyimide aerogels via freeze-drying, followed by vacuum impregnation of PEG into the aerogel matrix. The robust capillary forces generated by the hierarchically porous structure effectively immobilized the PCM, preventing leakage. The resulting composite exhibited exceptional thermal energy storage density (167.9 J/g) and outstanding flame retardancy.

## 5. Summary and Outlook

In this work, we review state-of-the-art development in controlled synthesis, surface modification, advanced molecular design, and composite engineering of polyimide materials for packaging and thermal management of electronic devices. Based on three core design strategies—molecular engineering, structural design, and functional modification, various thermophysical properties of polyimide materials, including hydrophobicity, chemical and friction resistance, dielectric constant and loss, thermal stability, thermal conductivity, mechanical properties, and EMI-shielding performances can be tuned and optimized to meet their application requirements. Recent years have witnessed exciting progress in realizing controlled synthesis and fabrication of polyimides in different forms, including solid thin films, fibers, porous foams, and achieving comprehensively enhanced thermophysical properties and functionalities, making them ideal candidates to address the challenges of next-generation electronic packaging and thermal management.

Continuous research efforts should be devoted to exploring controlled synthesis and fabrication of high-performance polyimides and their composites. The primary bottleneck in the large-scale production of polyimides lies in the inherent limitations of their precursor (PAA). It suffers from chemical instability and is prone to hydrolysis, which often leads to broader molecular weight distribution and degraded mechanical properties after imidization. Thermal sensitivity causes molecular chain crosslinking when stored at elevated temperatures. Additionally, the requirement for high-boiling-point polar solvents (e.g., NMP, boiling point 202 °C) results in solvent residues, and the volatilization of organic solvents introduces toxicity, conflicting with green manufacturing principles. Furthermore, traditional thermal curing necessitates stepwise heating from 80 °C to 300 °C for long durations (4–6 h), which is time- and energy-consuming. When preparing polyimide composites, the compatibility between fillers and the matrix has often been considered as a key factor affecting the integrity and performance of the obtained composites. In this regard, how to realize controlled mixing of fillers within polyimide matrix on a large scale remains a critical challenge to be addressed.

Future research should continue to integrate material innovation with application-oriented design to fully unlock the potential of polyimides in advanced thermal management systems and multifunctional electronic components. The development of these materials aligns perfectly with the evolving demands of 5G/6G technologies, flexible electronics, and high-power devices, offering new opportunities for groundbreaking advancements in the electronic industries. Moving forward, it is anticipated that electronic packaging and thermal management polyimide materials will advance toward smarter, more efficient, and more sustainable solutions, providing essential material support for the progression of information technology.

## Figures and Tables

**Table 1:** Solubility properties of modified polyimides [20].

Samples	DMF	DMAC	DMSO	NMP	Aecetone
FPI	+	+	+	+	+
CNFPI	++	++	++	++	-
CNOPI	++	++	++	++	-
CCNFPI	- -	- -	- -	- -	- -
CCNOPI	- -	- -	- -	- -	- -

++: soluble at room temperature; +: soluble on heating; -: partially soluble on heating; - -: insoluble; DMF, N,N-dimethylformamide; DMAC, dimethylsulfoxide; DMSO, dimethylsulfoxide; NMP, N-methylpyrrolidone.

**Table 2:** Polyimide composites with enhanced thermal conductivity and EMI shielding performance.

Filler	Loading (wt%)	Theral Conductivity (W/m·K)	SE (dB/mm)	Ref.
Ag/rGO	15% (Ag:rGO = 1:4)	2.12		[107]
CuNPs-CuNWs	10%	4.13		[106]
AlOOH nanowires	10%	3.568		[113]
Ag/BaFe_12_O_19_	15–20%	5	989	[130]
CF@NiCo	41%		80.6	[128]
CNT/Ag	CNT: 0.1% Ag: 12.4%	0.72	598	[135]
Vertical graphene nanosheets	20%	2.1	207.7	[133]
Graphene/carbon fiber	20%	1.65	36.5	[134]
Ti_3_C_2_Tₓ	60%		2466.7	[137]
M@GNS	15%	16.10		[116]
h-BN	30%	2.56		[119]
Mxene	40%	5.12		[141]
GO/BN	GO: 1% BN: 20%	11.203		[142]
rGO@CN	10%	6.08		[143]
